# The soil microbial food web revisited: Predatory myxobacteria as keystone taxa?

**DOI:** 10.1038/s41396-021-00958-2

**Published:** 2021-03-21

**Authors:** Sebastian Petters, Verena Groß, Andrea Söllinger, Michelle Pichler, Anne Reinhard, Mia Maria Bengtsson, Tim Urich

**Affiliations:** 1 Institute of Microbiology, Center for Functional Genomics of Microbes, University of Greifswald, Greifswald, Germany; 2 Department of Arctic and Marine Biology, UiT The Arctic University of Norway, Tromsø, Norway

**Keywords:** Next-generation sequencing, Transcriptomics, Microbial ecology

## Abstract

Trophic interactions are crucial for carbon cycling in food webs. Traditionally, eukaryotic micropredators are considered the major micropredators of bacteria in soils, although bacteria like myxobacteria and *Bdellovibrio* are also known bacterivores. Until recently, it was impossible to assess the abundance of prokaryotes and eukaryotes in soil food webs simultaneously. Using metatranscriptomic three-domain community profiling we identified pro- and eukaryotic micropredators in 11 European mineral and organic soils from different climes. Myxobacteria comprised 1.5–9.7% of all obtained SSU rRNA transcripts and more than 60% of all identified potential bacterivores in most soils. The name-giving and well-characterized predatory bacteria affiliated with the *Myxococcaceae* were barely present, while *Haliangiaceae* and *Polyangiaceae* dominated. In predation assays, representatives of the latter showed prey spectra as broad as the *Myxococcaceae*. 18S rRNA transcripts from eukaryotic micropredators, like amoeba and nematodes, were generally less abundant than myxobacterial 16S rRNA transcripts, especially in mineral soils. Although SSU rRNA does not directly reflect organismic abundance, our findings indicate that myxobacteria could be keystone taxa in the soil microbial food web, with potential impact on prokaryotic community composition. Further, they suggest an overlooked, yet ecologically relevant food web module, independent of eukaryotic micropredators and subject to separate environmental and evolutionary pressures.

## Introduction

Predation is a key process in structuring community composition in ecosystems and in maintaining biodiversity. Predator-prey interactions and dynamics among animals and consequences for ecosystem functioning have been studied extensively since the early days of ecology [1]. Predators can play such an impactful role in an ecosystem that a removal would result in a disruption of the food web, which is why they are considered as keystone taxa [1]. While less visible and thus less acknowledged, predation is not foreign to the microbial world. Eukaryotic and prokaryotic microorganisms are known to prey on other microorganisms in marine, aquatic, and terrestrial habitats as part of the microbial food web [2, 3].

Protists (single-celled heterotrophic eukaryotes) are traditionally considered the main microbial predators of bacteria. This notion stems from the fact that predation is a common lifestyle among protists while this lifestyle has been considered uncommon among bacteria. Predatory protists are known from both aquatic and soil environments and are a key component of the “microbial loop” responsible for the remineralisation of carbon and nutrients [2, 4]. Protist groups that are known to be able to feed on bacteria include *Amoebozoa*, *Cercozoa*, *Ciliophora*, *Euglenozoa*, *Foraminifera*, and *Heterolobosea*. Protists in aquatic environments have been relatively well characterized, both in terms of their identity and population size. Research in soils on the other hand has been much more hampered, since direct microscopic observation is challenging in the soil matrix, cultivation is often difficult, and available molecular tools have been biased and non-quantitative [5, 6].

Much fewer known prokaryotic species are considered predatory compared to eukaryotes, although a predatory lifestyle in prokaryotes probably evolved prior to its development in eukaryotes [7, 8]. Several bacterial predators have been identified, with more and more taxa exhibiting a predatory lifestyle being recognized recently. These include myxobacteria (the taxonomic position of which is subject to current discussions [9, 10], here still referred to as the order *Myxococcales*), *Lysobacter*, *Bdellovibrio* and like organisms, *Vampirococcus*, and *Daptobacter*, among others [7, 11–13]. Especially the myxobacteria, with their ‘wolf-pack hunting’ strategy, have been known as micropredators for more than 70 years and were isolated from soils world-wide [11, 14]. They have been divided into two suborders and eight families [15]. Diverse members of the myxobacteria have already been shown to be metabolically active in situ in the soil microbial food web [16].

Until recently, it has been impossible to assess both bacterial and protist community composition with the same methodology. Although PCR amplicon approaches targeting the 16S and 18S rRNA genes enabled the study of both groups separately, a direct comparison of their relative abundances was not possible due to the absence of universal primers that would tackle all groups without bias. Even though universal primer pairs are known from previous studies, they feature certain drawbacks that may select against 18S rRNA [17]. However, these obstacles can be circumvented when applying random hexamer-primed reverse transcription as in metatranscriptomics approaches that target SSU rRNA of organisms from all three domains of life [18]. Furthermore, these rRNA transcripts are indicative of ribosomes, not merely ribosomal genes. Thus, they are likely to be derived from metabolically active cells and can be considered markers for living biomass. The generated cDNA fragments originate from different regions of the SSU rRNA molecule unlike PCR primed specific sites, and are therefore insensitive to the presence of introns or primer mismatches. We have recently used this metatranscriptomic three-domain community profiling approach to reveal the diversity of the active soil protist communities within five different natural soil systems in Europe, including forest, grassland and peat soils as well as beech litter [5].

In this study, we aimed to broadly identify all soil micropredators using metatranscriptomics, including a yet understudied group—the predatory bacteria. We expected high abundances of myxobacteria in at least some soils, as found in previous studies [19, 20]. In fact, transcripts of potentially predatory bacteria, especially myxobacteria, were abundantly detected in all soils, while protist read abundances were much more variable and generally lower. The underlying causes and consequences for our perception of microbial predation in soils are discussed and an alternative model of the soil microbial food web is put forward.

## Materials and methods

### Data acquisition

The investigated 28 metatranscriptomes had been obtained from different previous studies on a range of European soils (see Table 1 for details and references). These included four samples from organic peatlands, three samples from organic floodplains, three samples from gleic fluvisols, three samples from mineral grasslands, two samples from organic forest litter, four samples from mineral forest soils, and three samples each from three different mineral shrubland soils. RNA, cDNA and sequences were obtained as previously described [18, 21–24].

**Table 1:** Context data for sampling sites.

Site	Abbreviation	Location	Climatic zone	Biome	Dominant vegetation	Soil type	Carbon content (%)	pH	Moisture (% soil dry weight)	# of replicates	Sampling time	Reference	Sequencing method
Peatland soil ”Knudsenheia”	PsK	Ny-Ålesund, Norway (Svalbard)	Arctic	Fen wet land	Mosses	Organic	>80	7.3	1010	2	August 2009	Tveit et al., 2013	454 GS FLX Titanium
Peatland soil ”Solvatn”	PsS	Ny-Ålesund, Norway (Svalbard)	Arctic	Fen wet land	Mosses	Organic	>80	7.6	900	2	August 2009	Tveit et al., 2013	454 GS FLX Titanium
Mofette	MO	Hartoušov, Czech Republic	Temperate	Floodplain	*Filipendula ulmaria*	Organic	22	4.7	N.A.	3	July 2013	Beulig et al., 2016	Illumina HiSeq 2500
Mofette reference	MR	Hartoušov, Czech Republic	Temperate	Floodplain	*Deschampsia cespitosa, Eriophorum vaginatum*	Organic	23	5.3	N.A.	3	July 2013	Beulig et al., 2016	Illumina HiSeq 2500
Rothamsted grassland	RS	Rothamsted, United Kingdom	Temperate	Grassland	N.A.	Mineral	<3	4.9	33	2	July 2009	Geisen et al., 2015	454 GS FLX Titanium
Rotböhl	RB	Darmstadt, Germany	Temperate	Grassland	N.A.	Mineral	<2	7.1	32	1	January 2006	Urich et al., 2008	454 GS 20
Forest Litter	FL	Vienna woods, Austria	Temperate	Temperate deciduous forest	*Fagus sylvatica*	Organic	N.A.	N.A.	18	2	May 2008	Geisen et al., 2015	454 GS FLX
Forest Soil	FS	Vienna woods, Austria	Temperate	Temperate deciduous forest	*Fagus sylvatica*	Mineral	8	4.5–5.1	43–64	4	May 2008	Geisen et al., 2015	454 GS FLX
Mine L	MiL	Coto Txomin, Spain	Temperate	Shrubland	*Ulex europaeus*	Mineral	2.53	3.9	52	3	March 2011	Epelde et al., 2015	Illumina HiSeq 2000
Mine M	MiM	Coto Txomin, Spain	Temperate	Shrubland	*Festuca rubra*	Mineral	9	5.6	49	3	March 2011	Epelde et al., 2015	Illumina HiSeq 2000
Mine H	MiH	Coto Txomin, Spain	Temperate	Shrubland	*Festuca rubra*	Mineral	3.1	5.9	30	3	March 2011	Epelde et al., 2015	Illumina HiSeq 2000

### Bioinformatic analysis

Raw sequence datasets were filtered to a minimum length of 200 nucleotides and a minimum mean quality score of 25 using prinseq-lite [25]. SSU rRNA sequences were identified via SortMeRNA [26]. USEARCH [27] was used to randomly subsample datasets to a maximum of 100,000 sequences. The datasets were mapped against the CREST database silva123.1 by blastn [28, 29]. The obtained blastn files were taxonomically analyzed using MEGAN [30] (min score 155; top percent 2.0; min support 1). The number of SSU rRNA reads of the investigated organisms was normalized to total read counts. Investigated taxa with predatory lifestyle were *Myxococcales*, *Bdellovibrionales*, *Lysobacter*, *Daptobacter*, and *Vampirococcus* as prokaryotic micropredators. Among eukaryotic organisms we considered the protist groups *Amoebozoa*, *Cercozoa*, *Ciliophora*, *Foraminifera*, *Euglenozoa,* and *Heterolobosea* and also some *Nematoda* as potential micropredators (see below). Among myxobacteria, the non-predatory genus *Sorangium* was excluded [11, 31, 32]. Among *Nematoda*, the orders *Araeolaimida*, *Chromadorida*, *Desmodorida*, *Enoplida*, *Monhysterida*, *Rhabditida*, and *Triplonchida* were considered bacterial-feeding, according to Yeates et al. [33]. The read counts of the analyzed bacterial micropredators where subtracted from the total bacterial SSU rRNA and the remainder of counts were considered prey bacterial rRNA. The read counts of each analyzed bacterivorous group were then normalized to the prey bacterial SSU rRNA reads.

Total community SSU rRNA data from organic, excluding mofette (MO) samples due to being suboxic und less comparable, and mineral soils were tested for differentially abundant sequences with the R package edgeR ([34]; functions glmFit and glmLRT), using non-normalized total read counts. Organic soils were defined according to carbon content [35].

### Predation assays

The myxobacteria *Haliangium ochraceum*, *Stigmatella aurantiaca*, *Kofleria flava*, *Corallococcus coralloides,* and *Chondromyces robustus* were purchased from the German Collection of Microorganisms and Cell Cultures (DSMZ, Table 2). *M. fulvus* was isolated from soil in Greifswald. The potential prey bacteria were taken from the Bacterial Strain Collection of the University of Greifswald (SBUG, Table 2). *H. ochraceum* was cultivated on VY/4-SWS-agar and *K. flava* and *C. robustus* were cultivated on VY/2-agar, as suggested by DSMZ. *C. coralloides, M. fulvus*, and *S. aurantiaca* were cultivated in SP, CY, or VY/2 liquid medium, as suggested by DSMZ. Agar plates were incubated for 7–10 days and liquid cultures for 4 days in a horizontal shaker (Multitron, INFORS HT) at 170 rpm. Potential prey bacteria were cultivated on LB Agar or nutrient agar for 3 days. All incubations were performed at 30 °C.

**Table 2:** Myxobacteria and potential prey organisms used in predation assays.

*Myxobacteria*		
Deltaproteobacteria		*Myxococcus fulvus* (SBUG 2153)
		*Corallococcus coralloides* (DSM 2259)
		*Haliangium ochraceum* (DSM 14365)
		*Chondromyces robustus* (DSM 14608)
		*Kofleria flava* (DSM 14601)
		*Stigmatella aurantiaca* (DSM 17044)
*Potential prey bacteria*		
Gram-positive	Firmicutes	*Bacillus megaterium* (SBUG 518)
		*Bacillus subtilis* (SBUG 14)
	Actinobacteria	*Gordonia rubripertincta* (SBUG 1971)
		*Micrococcus luteus* (SBUG 16)
Gram-negative	Betaproteobacteria	*Cupriavidus basilense* (SBUG 290)
		*Delftia acidovorans* (SBUG 1233)
	Gammaproteobacteria	*Enterobacter cloacae* sp. *dissolvens* (SBUG 2043)
		*Escherichia coli* K12 (SBUG 1135)
		*Trabulsiella guamensis* (SBUG 2045)
		*Pseudomonas fluorescens* (SBUG 1177)
		*Pseudomonas putida* mt-KT2440 (SBUG 2042)
		*Pseudomonas stutzeri* (SBUG 93)

Predation assays were done according to Müller et al. [36], by co-cultivation of the respective myxobacterium with one potential prey organism in petri dishes. Assays were performed on 12.5 ml agar plates with medium specific for the myxobacterium or on 0.5% bacto peptone agar in case of *C. coralloides* and *M. fulvus* [36]. One full inoculation loop of prey bacteria was taken from an agar plate and suspended in 233 µl of 0.9% sodium chloride solution. Avoiding bubbles, 50 µl of the suspension were applied to the middle of the agar plate. After drying, a 0.1 cm^2^ agar piece from a myxobacteria plate (in case of *H. ochraceum, K. flava* and *C. robustus*) were applied upside down to the center of the prey drop. For liquid cultures (in case of *M. fulvus, C. coralloides*, and *S. aurantiaca*) 10 µl were applied to the center of the prey drop. Subsequently, the plates were incubated at 30 °C. Quality of potential prey lysis was monitored repeatedly over the course of 14 days. Clearly visible lysis >= 1 mm in diameter where the prey organism had disappeared visibly was considered positive. No clearly visible lysis was considered negative. Experiments were repeated two to four times (see Supplementary Table 1).

## Results and discussion

### Metatranscriptomics-enabled census of potential soil micropredators

**Fig. 1:**
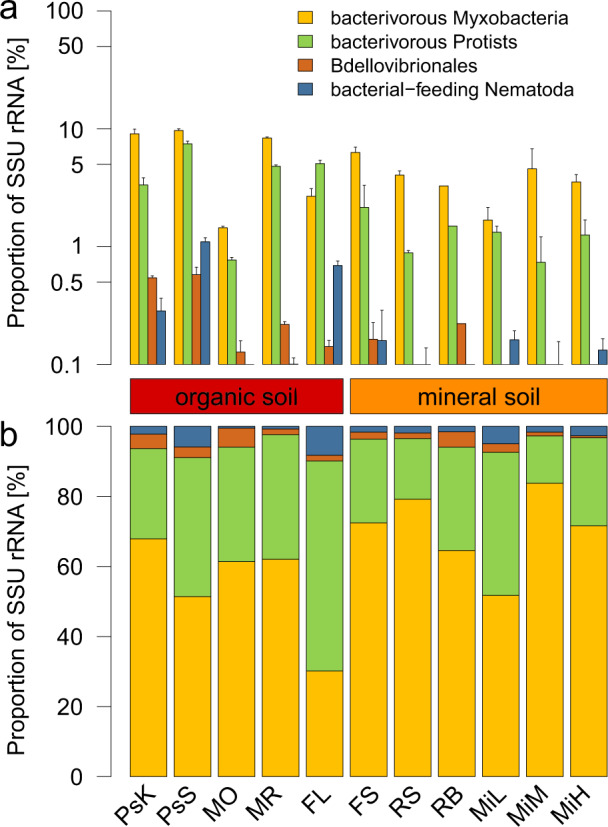
Screening of pro- and eukaryotic micropredators in soils. **a** Proportion of major identified micropredator SSU rRNA normalized to total SSU rRNA. **b** Proportion of major identified micropredator SSU rRNA normalized to total micropredator SSU rRNA. Error bars show standard deviation of replicates. For sites see Table 1.

The soil microbial food web is crucial for carbon and nitrogen cycling in soils [2, 37–39]. Several functional guilds have been described that belong to both pro- and eukaryotic domains of life, such as saprotrophic bacteria and fungi, as well as predatory protists and nematodes as major consumers of bacteria [4, 40–43]. This functional and phylogenetic complexity makes identification of the players by molecular methods challenging. The rRNA fraction of metatranscriptomics data enables broad three-domain community profiling [18, 44–46]). Although rRNA transcripts are not directly equivalent to organismic abundances, for example as determined by microscopy, they offer a broad and relatively unbiased view onto soil microbial communities. To shed light on the functional groups in the soil microbial food web, we screened the SSU (16S and 18S) rRNA fraction of 28 soil metatranscriptome datasets from 11 different soils across Europe (Table 1) for known bacterivorous pro- and eukaryotes. In all investigated soils the myxobacteria comprised a relatively high proportion of the overall SSU rRNA transcripts. This confirms a recent PCR/16S rRNA gene based survey where myxobacteria also covered a substantial fraction (1.5–4.7%, [47]; 4.1%, [19]). On our study, they ranged from a fraction of 1.5–9.7% of all SSU rRNAs in the individual soils (4.9% of total SSU rRNAs across all datasets), which was higher than that of all other investigated bacterivores (Fig. 1a). Their highest proportion in relation to total SSU rRNA was detected in an organic fluvisol (MR) and in peat soils (PsK, PsS). A beech litter (FL) was the only exception in the pattern, i.e., here the predatory protists were the most abundant bacterivorous group, in terms of SSU rRNA transcripts. Comparing all investigated bacterivorous groups, the proportion of myxobacteria SSU rRNAs was more than 60% of all micropredators in eight of the eleven sites, including most mineral soils (Fig. 1b). It was only in the forest litter that their proportion was below 30%. Overall, SSU rRNAs of predatory protists were the second most abundant amongst the investigated potential micropredators (Fig. 1). Their abundance ranged from 0.4% in mineral soil to 7.7% of all SSU rRNA transcripts in organic peatland (Fig. 1a). In all the sampled sites (except the forest litter sample FL), the proportion of predatory protists reads was not more than 40% of all investigated potential micropredator SSU rRNA (Fig. 1b).

### Poorly characterized myxobacteria dominate bacterial micropredators

**Fig. 2:**
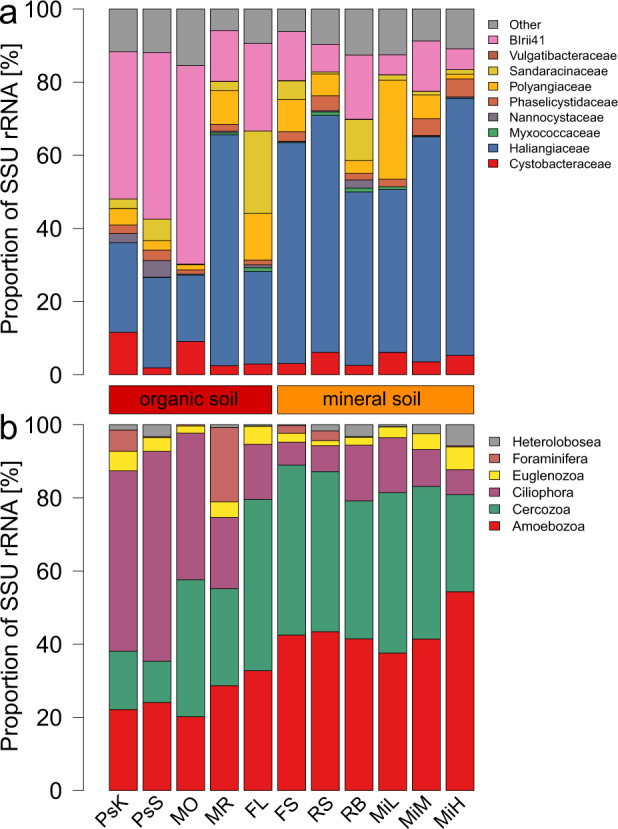
Screening of *Myxococcales* and predatory protist taxa. **a** Proportion of identified myxobacteria families SSU rRNA normalized to overall *Myxococcales* SSU rRNA. **b** Proportion of predatory protist SSU rRNA normalized to total predatory protist SSU rRNA. For sites see Table 1.

Although the myxobacteria comprised a rather constantly high proportion of bacterial SSU rRNA in all sites, differences at family-level composition were observed among the soils (Fig. 2a). However, the most dominant families in terms of SSU rRNA reads were *Haliangiaceae* and *Polyangiaceae*, similar to what has been observed before (e.g., [19, 43]). Abundant was also the Blrii41 clade, a family-level group in the SILVA taxonomy that is currently devoid of cultured representatives (Fig. 2a). These three groups comprised more than 2/3 of all myxobacteria SSU rRNAs in all but one site. *Haliangiaceae* and *Polyangiaceae* rRNAs were more abundant in mineral soils, while Blrii41 rRNAs were more abundant in organic soils. Interestingly, SSU rRNAs of the name-giving family *Myxococcaceae*, which contains, among others, the most frequently isolated and well characterized micropredators of the genera *Myxococcus* and *Corallococcus* [11, 36, 48–59], were barely present. The *Myxococcaceae* are known to be easily cultivable from a variety of environmental samples. This is indicative of a strong cultivation bias within the myxobacteria, as previously observed for many other groups of bacteria and archaea (e.g., [60, 61]), and show that other, less well-characterized families are in fact much more abundant in soil. Few studies on their prey spectrum have been conducted. Members of the families *Nannocystaceae* and *Phaselicystidaceae* are able to lyse bacterial cells [62, 63]. In addition, lysis of bacteria and yeasts has been reported for members of *Cystobacteraceae*, *Haliangiaceae*, *Kofleriaceae*, and *Polyangiaceae* [15, 32, 62–66]. The taxonomy within the myxobacteria is in constant movement [15, 32, 62–66] and may well cause confusion when analyzing 16S rRNA data. For instance, the family *Haliangiaceae* has no standing valid nomenclature, but is widely used as taxonomic entity. In the SILVA taxonomy used here, it comprises a broad clade, including the validly described family *Kofleriaceae* [65] a term which is often used synonymously.

The *Haliangiaceae* are particularly interesting myxobacteria due to their high abundance. In the applied taxonomy (SILVA) the *Haliangiaceae* clade is very broad and includes the genus *Kofleria* as well as many uncultivated phylotypes. However, only three species are currently validly described, *Haliangium ochraceum* and *H. tepidum*, as well as *Kofleria flava* [64, 65]. Thus, it is important to investigate the biology of those highly abundant but less characterized myxobacteria, especially regarding their predatory lifestyle and prey spectrum.

The *Bdellovibrionales* comprised lower SSU rRNA abundances, below 10% of the investigated potential micropredators, in all sampling sites (Fig. 1). Similar to the myxobacteria, their highest relative abundance was observed in organic soils (0.6% of the total SSU rRNA in peatland soil, Fig. 1). *Bdellovibrio* species feature comparably narrow prey range, with their small cells preying exclusively Gram-negative bacteria. Cells from most species are specialized on entering their prey as periplasmic parasites [13]. *Lysobacter*, a genus known to be able to control a variety of plant and animal pathogens, comprised the lowest SSU rRNA abundances of all detected micropredators (<0.1%; data not shown). Finally, we did not detect *Vampirococcus* and *Daptobacter* reads in any of the investigated samples.

### Eukaryotic micropredators vary strongly depending on soil type

Members of the *Amoebozoa*, *Cercozoa*, and *Ciliophora* were the three most abundant bacterivorous groups of protists, (Fig. 2b), as previously found [21]. While *Amoebozoa* and *Cercozoa* reads dominated in mineral soils, the *Ciliophora* reads were most abundant in organic soil, possibly due to the higher moisture of these soils. The remaining predatory groups *Foraminifera*, *Euglenozoa*, and *Heterolobosea* accounted for low read abundances on average. The mofette reference (MR) samples were an exception, with *Foraminifera* comprising more than 20% of SSU rRNA reads.

Among nematodes, on average 38% were classified as bacterivorous (Supplementary Fig. 1). Among them, the orders *Monhysterida*, *Rhabditida*, and *Triplonchida* were particularly abundant. In organic soils, especially in arctic peat soils, their fraction was comparably higher than in mineral soils. They showed greater variation in abundance compared to the aforementioned taxa, especially in organic soils, where they showed both their highest abundance (1.2% in PsS), and also their lowest abundance (<0.01% in MO). In addition, all sites had *Nematoda* SSU rRNA below 10% of all micropredator SSU rRNA (Fig. 1b). Their highest proportions occurred in the organic forest litter samples (FL) and their lowest proportions in samples from the mofette soil (MO).

### Predation assays of understudied myxobacteria reveal broad prey spectrum

**Fig. 3:**
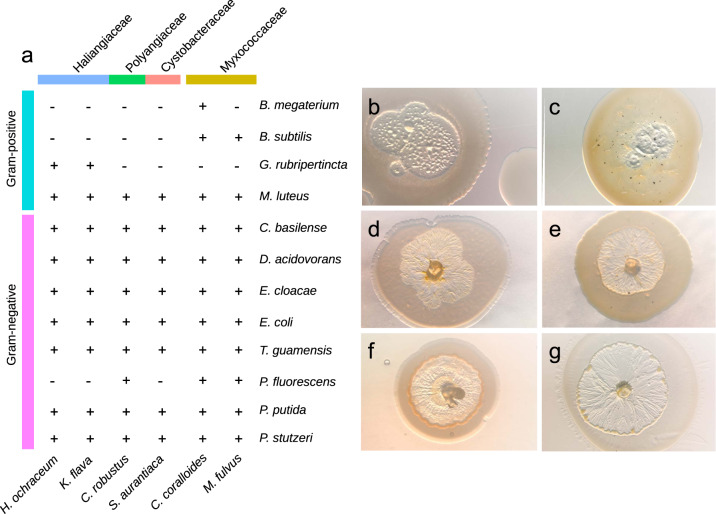
Prey spectrum of myxobacteria. **a** Overview of lysis in predation assays. + Visible lysis ≥ 1 mm in diameter. - No clear lysis after 14 days. **b** Lysis of *C. basilensis* colony by *M. fulvus* (7 days) (**c**) Lysis of *M. luteus* colony by *M. fulvus* (7 days) (**d**) Lysis of *E. coli* colony by *H. ochraceum* (5 days) (**e**) Lysis of *M. luteus* colony by *H. ochraceum* (7 days) (**f**) Lysis of *P. putida* colony by *C. robustus* (4 days) (**g**) Lysis of *D. acidovorans* colony by *K. flava* (4 days).

There is a good body of literature on the broad prey spectrum of the genera *Myxococcus* and *Corallococcus* of the *Myxococcaceae* family [56–58]. However, much less is known about the predatory potential and prey spectrum of the abundant families detected in this study (Fig. 2a). For instance, the three characterized species of *Haliangiaceae*/*Kofleriaceae* are only known to lyse *E. coli, M. luteus*, and yeast cells [64, 65], but no prey spectrum has been recorded of this most abundant soil group. To shed light on the prey spectrum of *Haliangiaceae*, *Polyangiaceae*, and *Cystobacteraceae*, predation assays with 12 potential prey bacteria were performed and compared to two *Myxococcaceae* strains (Table 2 and Fig. 3). As previously described [36, 52–59], *M. fulvus* and *C. coralloides* had broad prey spectra in the assays, with a better lysis of Gram-negative than Gram-positive bacteria (Fig. 3). Similarly broad prey spectra were detected with *H. ochraceum and K. flava* (*Haliangiaceae*/*Kofleriaceae*)*, S. aurantiaca* (*Polyangiaceae*), and *C. robustus* (*Cystobacteraceae*). Again, Gram-negative bacteria were more efficiently lysed than Gram-positive strains, although with variations among the tested myxobacteria. *H. ochraceum* and *K. flava* were both capable of lysing nine of the twelve prey bacteria. They were the only ones capable of lysing the Gram-positive actinobacterium *G. rubripertincta*. *C. robustus* and *S. aurantiaca* lysed nine and eight of the prey strains, respectively and had a very similar prey spectrum. In summary, the four understudied myxobacteria were identified as potent micropredators in the plating assays, similar to members of the *Myxococcaceae*. Furthermore, the six recorded predation spectra combined result in a gapless predation spectrum, where all potential prey bacteria were subject to lysis by at least one myxobacterium.

Yet, one must be aware that the experimental design is highly artificial under the described laboratory conditions and featuring only one potential predator and one potential prey organism. The assays should be interpreted carefully, since they cannot simulate predation on and in complex environmental microbial communities. Although in vitro assays should not be directly extrapolated to the conditions in situ, in the soil matrix, our findings provide the best data currently available and the results show a high predatory potential of the understudied families *Haliangiaceae*/*Kofleriaceae*, *Polyangiaceae*, and *Cystobacteraceae*. Together with their remarkably high abundance in different soils this indicates a substantial role as micropredators of bacteria in the soil microbial food web.

However, it should be noted that the micropredator abundance data in this study are all derived from the abundance of SSU rRNA reads in metatranscriptomes. This does not directly reflect organismic abundance but is rather considered a proxy of living biomass [67]. Also, we are aware that predation is effective on cellular and not ribosomal level. Consequently, the importance of predators cannot directly be derived from rRNA abundances. For instance, few nematodes might consume lots of bacteria [68, 69]. Moreover, several factors need to be taken into account when comparing the SSU rRNA from different pro- and eukaryotic organisms. Results of various studies suggest differences in RNA contents per biomass, (1) between organisms and (2) between growth phases, respectively [70–74]. The RNA content of *E. coli* was determined to be 15.7% of dry mass (dm) [70], of *Bacillus subtilis* between 8.5 and 14% dm^−1^ [71], *Saccharomyces cerevisiae* 23% dm^−1^ [72], *Aspergillus* 5.9% [73], and *Penicillium chrysogenum* between 5 and 8% [74]. Furthermore, prokaryotic cells are known to contain more RNA while in an exponential phase than cells in their stationary phase (e.g., [71]). Preliminary data (Petters and Urich, unpublished) suggest that correction factors need to be applied when comparing rRNA-based abundances from metatranscriptomes between pro- and eukaryotes. Also, RNA extraction efficiency might vary between different groups of organisms.

### Differences in food webs between mineral and organic soils

**Fig. 4:**
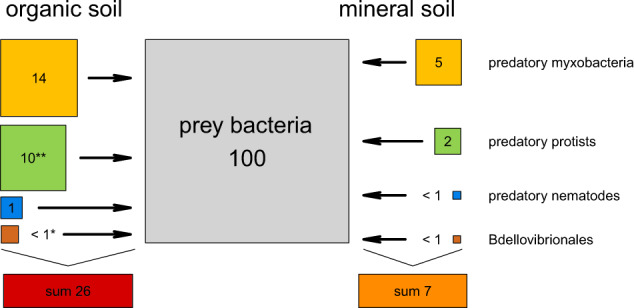
Comparison of organic and mineral soils. Predator:prey ratio of major identified micropredator SSU rRNA normalized to SSU rRNA of prey bacteria. Average in organic soils (excluding MO samples) on the left; average in mineral soils on the right. Area of boxes is proportional to abundance of SSU rRNA. Numbers show predator:prey ratios of micropredator SSU rRNA. *Lysobacter* data are not shown due to low abundances. Groups were tested for differentially expressed sequences (*p < 0.05; **p < 0.01).

To shed light on effects of soil physico-chemical parameters such as carbon content, we compared micropredator abundance between mineral and organic soils (Fig. 4). In organic soils total micropredator SSU rRNAs equaled 26% of potential prey bacteria, as compared to only 7% in mineral soils. We want to point out that the classification of all non-predatory bacteria as “prey bacteria” is a simplification, since certain species likely are protected from predation, e.g., due to the production of spores or antibiotics. Myxobacteria SSU rRNA reads comprised the highest micropredator proportion in both soil types, 14% of prey bacteria in organic soils, and 5% in mineral soils. This difference in abundance was not significant (*p* = 0.36). The SSU rRNA abundances of predatory protists were almost equally abundant as myxobacteria in organic soils (10%), but only 2% in mineral soils. This difference in abundance was significant (*p* < 0.01). *Bdellovibrionales* were generally much less abundant (below 1%) and showed significant differences (*p* = 0.01) between both soil types. While rather abundant in organic soils (1%), nematodes were less abundant in mineral soils (below 1%), however, this difference was not statistically significant (*p* = 0.78). It is remarkable that lower micropredator abundances were detected in mineral soils as compared to organic soils. This may be due to less available carbon in mineral soils resulting in lower prey cell density. In addition, the smaller pore sizes in mineral soils might provide restricted access for protists to their bacterial prey, as compared to the smaller cells of myxobacteria. Soil type is already known to affect the composition and ratio of smaller and larger predators [75, 76]. Moreover, it has been suggested that different pore sizes in soils are main drivers of compartmentalization of different prey and predator organisms [77]. Thus, microorganisms inhabiting non-continuous capillary pores could be protected from predation by protists and *Nematoda*, but not from the similarly-sized myxobacteria. The prokaryotes inhabiting the organic soil horizons, with unprotected macro-pore space, would in turn be subjected to higher grazing pressure.

### Predatory myxobacteria as keystone taxa in the microbial food web?

Traditionally, protists are considered to be the dominant group preying on bacteria (e.g., [78, 79]). In contrast to this, our data suggest an importance, possibly even dominance of myxobacteria as soil predators. In fact, myxobacteria comprised approximately three quarters of all micropredators in mineral soils. The myxobacteria and protists exhibit fundamentally different predation strategies, with the much smaller myxobacteria being known for their social ‘wolf-pack’ hunting combined with the secretion of lytic enzymes, as compared to the larger phagotrophic protists [11]. The more similar cell size of myxobacteria and prey bacteria could thus favour myxobacterial predation in mineral soils with small pores. Still, also some amoebae are able to extend their pseudopodia into small pores. Furthermore, one must be aware that the samples were merely a selection of soils from different studies processed with a variety of different sequencing platforms and thus our findings should be investigated on a wider scale.

**Fig. 5:**
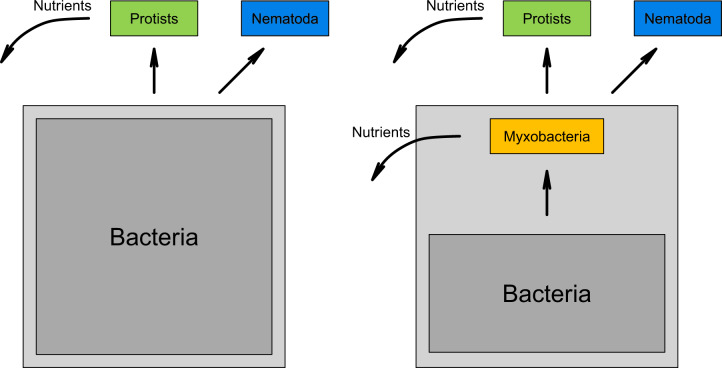
Simplified soil microbial food web. Left: Traditional microbial food web with separate roles of prokaryotic and eukaryotic organisms. Right: Microbial food web containing a separate module, independent of eukaryotic organisms. Straight arrows: links between trophic levels. Bent arrows: release of nutrients.

Keystone taxa have a disproportionate effect on their surrounding environment, and are often predators [1, 80]. Identifying keystone taxa in microbial communities is not trivial, as direct interactions can usually not be observed [80, 81]. However, given the broad prey range of myxobacteria in vitro and their high abundance in situ [19] in different kinds of soils suggest a major influence on structuring the prokaryotic community composition, and might warrant their classification as keystone taxa (Fig. 5). However, this remains to be shown in future studies. In fact, our study did not provide direct proof of whether the myxobacteria (or any presumed micropredator) actually showed bacterivorous behavior in situ. There is direct in situ evidence for myxobacterial bacterivory from RNA-stable isotope probing studies [16, 43].

Thus, we and others show that myxobacteria can prey on a broad range of bacteria (e.g., [16, 56, 82]). These observations hint at the presence of an ecologically important additional module in the microbial food web, i.e., trophic interactions among the bacteria and independent of protists and nematodes, that has hardly been taken into consideration until today (Fig. 5). Since these interactions are independent of eukaryotic micropredators this module might be subject to separate environmental and evolutionary pressures.

Although rather speculative, (myxo)bacterial micropredators might not only be important for shaping microbial communities, but might also be relevant for the recycling of nutrients in soils, as has been shown for protists, i.e., in the microbial loop [4, 83]. Clearly, more studies are needed to fully understand the role of these fascinating microorganisms in the microbial food web.

## Supplementary information

Supplementary Table 1

Supplementary Figure 1
