# Supplementary material for: The soil microbial food web revisited: Predatory myxobacteria as keystone taxa?
Source: ISME J. 2021 Mar 21;15(9):2665–75. doi: 10.1038/s41396-021-00958-2 (PMC8397742; doi:10.1038/s41396-021-00958-2)
Supplement: Supplementary file 1 — Supplementary Table 1 [file 41396_2021_958_MOESM1_ESM.docx]

**Supplementary Table 1.** Number of replicates per specific predator-prey combination

|  | *H. ochraceum* | *K. flava* | *C. robustus* | *S. aurantiaca* | *C. coralloides* | *M. fulvus* |
| --- | --- | --- | --- | --- | --- | --- |
| *B. megaterium* | 2 | 4 | 4 | 4 | 2 | 4 |
| *B. subtilis* | 2 | 4 | 4 | 4 | 2 | 4 |
| *G. rubripertincta* | 2 | 4 | 4 | 4 | 2 | 4 |
| *M. luteus* | 3 | 4 | 4 | 4 | 2 | 4 |
| *C. basilense* | 4 | 4 | 2 | 4 | 2 | 2 |
| *D. acidovorans* | 4 | 4 | 4 | 4 | 2 | 4 |
| *E. cloacae* | 2 | 4 | 4 | 4 | 2 | 4 |
| *E. coli* | 3 | 4 | 4 | 3 | 4 | 4 |
| *T. guamensis* | 4 | 4 | 2 | 2 | 2 | 4 |
| *P. fluorescens* | 2 | 2 | 2 | 2 | 2 | 2 |
| *P. putida* | 3 | 4 | 4 | 4 | 2 | 2 |
| *P. stutzeri* | 2 | 4 | 4 | 4 | 2 | 4 |
