# Supplementary figures and images for: The soil microbial food web revisited: Predatory myxobacteria as keystone taxa?
Source: ISME J. 2021 Mar 21;15(9):2665–75. doi: 10.1038/s41396-021-00958-2 (PMC8397742; doi:10.1038/s41396-021-00958-2)

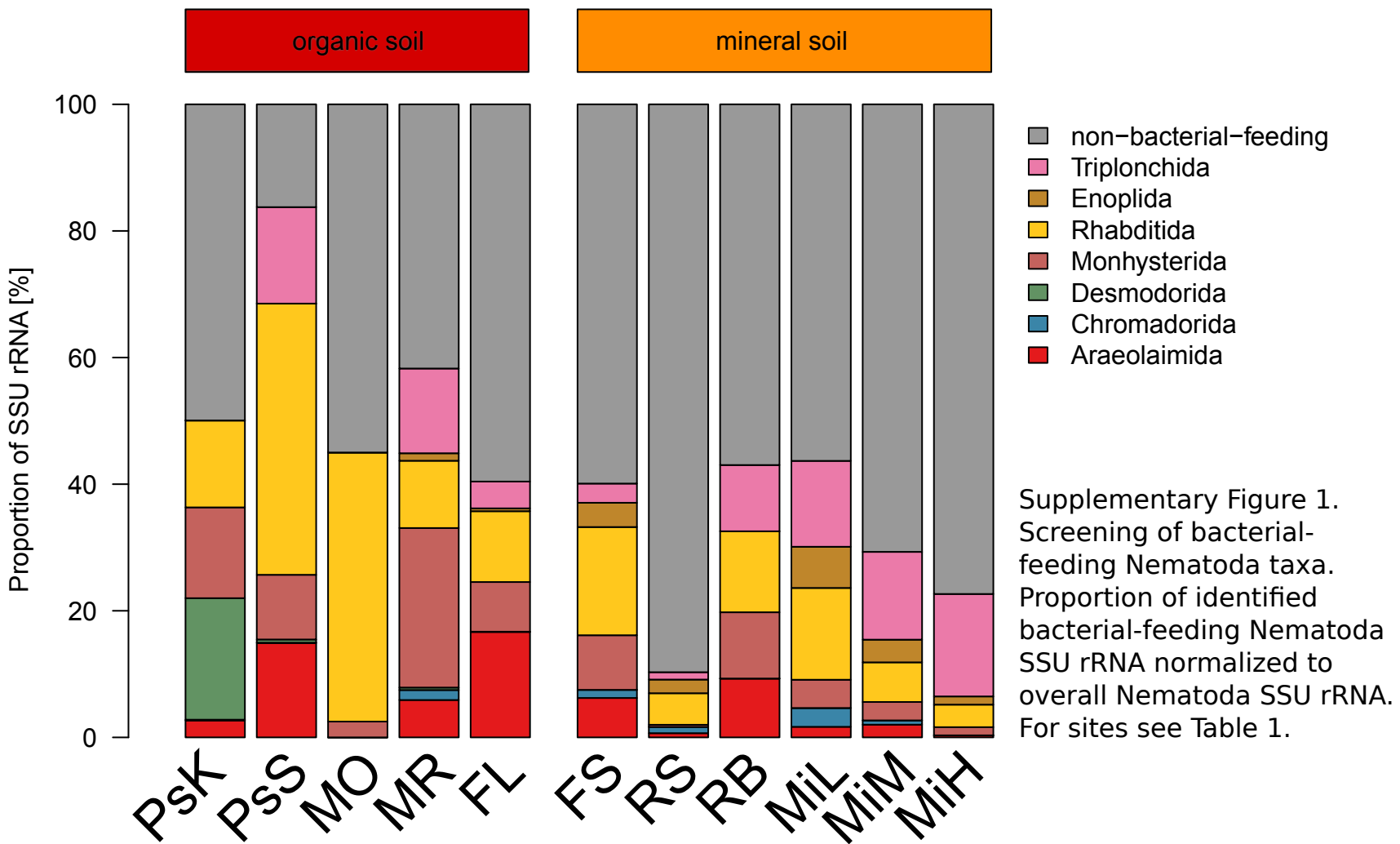

Supplement: Supplementary file 2 — Supplementary Figure 1 [file 41396_2021_958_MOESM2_ESM.pdf]
